# Typology of person-environment fit constellations: a platform addressing accessibility problems in the built environment for people with functional limitations

**DOI:** 10.1186/s12889-015-2185-4

**Published:** 2015-09-02

**Authors:** Björn Slaug, Oliver Schilling, Susanne Iwarsson, Gunilla Carlsson

**Affiliations:** Department of Health Sciences, Faculty of Medicine, Lund University, Box 157 SE-221 00, Lund, Sweden; Department of Psychological Ageing Research, Institute of Psychology, University of Heidelberg, Heidelberg, Germany

## Abstract

**Background:**

Making the built environment accessible for all regardless of functional capacity is an important goal for public health efforts. Considerable impediments to achieving this goal suggest the need for valid measurements of acccessibility and for greater attention to the complexity of person-environment fit issues. To address these needs, this study aimed to provide a methodological platform, useful for further research and instrument development within accessibility research. This was accomplished by the construction of a typology of problematic person-environment fit constellations, utilizing an existing methodology developed to assess and analyze accessibility problems in the built environment.

**Methods:**

By means of qualitative review and statistical methods we classified the person-environment fit components covered by an existing application which targets housing accessibility: the Housing Enabler (HE) instrument. The International Classification of Functioning, Disability and Health (ICF) was used as a conceptual framework. Qualitative classification principles were based on conceptual similarities and for quantitative analysis of similarities, Principal Component Analysis was carried out.

**Results:**

We present a typology of problematic person-environment fit constellations classified along three dimensions: 1) accessibility problem range and severity 2) aspects of functioning 3) environmental context. As a result of the classification of the HE components, 48 typical person-environment fit constellations were recognised.

**Conclusions:**

The main contribution of this study is the proposed typology of person-environment fit constellations. The typology provides a methodological platform for the identification and quantification of problematic person-environment fit constellations. Its link to the globally accepted ICF classification system facilitates communication within the scientific and health care practice communities. The typology also highlights how relations between aspects of functioning and physical environmental barriers generate typical accessibility problems, and thereby furnishes a reference point for research oriented to how the built environment may be designed to be supportive for activity, participation and health.

## Introduction

For health promotion and public health efforts, it is vitally important to foster physical environments supportive of activity, participation and health [1]. One aspect which deserves specific attention in this regard concerns the relationship between the person and the built environment [2]. In particular, this relationship concerns the degree to which access to utilities in the environment is facilitated or obstructed, depending on the functional capacity of individuals. Due to the rapidly ageing population more people will live for a longer period of their lifes with limited functional capacity. Therefore, accessibility issues can be expected to gain in importance for societal policies concerning the built environment. According to internationally approved policies [3, 4], housing, public buildings and public transportation should be accessible for all citizens, regardless of functional capacity. Nevertheless, serious deficiencies can still be observed, even in countries where legislation is characterized by foresightedness with regard to accessibility problems. To rectify these shortcomings calls for a great variety of measures to be undertaken, but a fundamental condition for such measures to be adequate and efficient is that systematic and objective methods for problem identification are utilized. With the use of reliable and valid methods, the conditions for achieving the goal of making the built environment accessible for all could be improved [5–7].

Theoretically, the concept of accessibility is underpinned by the ecological theory of ageing (ETA) [2], also referred to as the competence-press or person-environment fit model. The ETA defines the person in terms of a set of competencies, and the environment in terms of its demands, labelled environmental press. With the addition of the docility hypothesis [8], stating that those with lower personal competencies are more vulnerable to environmental press whereas those with higher competencies can withstand greater environmental press, the ETA has become one of the most influential person-environment fit theories. Based on the ETA and originating from the so-called Enabler Concept [9], an internationally recognized and research-based methodology for assessing and analyzing accessibility problems in housings [10] is now well established. The Enabler methodology (EM) treats accessibility as a quantifiable person-environment fit measurement, where the personal component consists of functional limitations and the environmental component of barriers in the physical environment. It is thus a composite measurement attaching varying degrees of severity to different person-environment fit constellations [10]. Thereby the measurement permits an analysis that is detailed as well as sensitive to any variation in either of the two components. Methodological research results indicate good predictive value for this measurement, where varying characteristics of the personal component produce different sets of improvement priorities [11, 12]. For example, for groups where limitations in movement are prevalent, the measurement particularly indicates environmental barriers that obstruct mobility as prioritized for removal. Up to now however, the EM has mainly been applied to the housing environment [13]. In order to explore the potential for valid extension of the EM to other environmental arenas, this study aimed to provide a methodological platform useful for further research and instrument development within the field of accessibility research. More specifically, we constructed a typology of problematic person-environment fit constellations, addressing accessibility problems in the built environment for persons with functional limitations.

## Background

### The enabler methodology

The principal idea of the EM is that a quantitative measurement of accessibility problems can be produced by juxtaposing systematic and structured checklists of environmental features with certain personal characteristics. The environmental checklist is comprehensive in coverage of features that are potentially limiting or hindering access in a given context, such as narrow door openings, high thresholds, absence of handrails etc. The checklist of personal characteristics is delimited to limitations in the functional capacity of an individual [14], such as poor balance, incoordination, limitations of stamina etc., relevant for activities implying access to the environment. Considered as indicators of more severe functional limitations, the personal component also includes use of mobility devices. When juxtaposing the two checklists, the intersections between each personal characteristic and each environmental feature are assigned pre-defined scores on a scale, grading the severity in terms of accessibility problems. For a description of the process of how the severity scores were originally defined by means of expert panels, see [9]. By summing up the scores, an aggregate measure is computed, representing the magnitude of accessibility problems in a particular case [10]. Based on the original scoring of the Enabler Concept [9], the scoring has been successively validated over the years, taking advantage of the results of empirical research as well as expert opinions from different professions, such as occupational therapy and architecture [13].

This methodology has been successfully applied in an instrument targeting accessibility of the housing environment (which is here considered as one environmental arena), the Housing Enabler (HE) [10]. That means one essential element needed for an extension of the methodology to other environmental arenas is already in place. That is, the HE checklist for identification of functional limitations is appropriate regardless of the environmental arena. On the other hand, the HE checklist for environmental barriers only covers the housing environment arena, and to adequately assess for example public buildings, shopping malls, theaters, bus stops etc., new lists of barriers have to be compiled (see e.g. [15]). Moreover, to establish valid scores of severity when juxtaposing each new environmental barrier to each functional limitation is challenging and requires additional methodological efforts. This is one of the most important reasons why despite of considerable research efforts throughout several years (see e.g. [16]), results are still not comprehensive enough for a valid extension of the EM to other environmental arenas. As a methodological approach that could provide a valid basis for a scoring rationale in a simplified manner, we therefore opted for the construction of a classification system. That is, to sort out and classify the dimensions essential to capture accessibility problems on a general level. The severity scores for new environmental barriers could then ideally be established just by finding its proper classification.

## Methods

### Typology construction as a methodological approach

A multi-dimensional classification system based on conceptual similarities is commonly referred to as a typology [17]. As dimensions essential to capture accessibility problems we considered: 1) the range and severity of problems generated, 2) the implied aspects of functioning of the individual (vision, mobility etc.), and 3) the environmental contexts where the barriers may occur (the kitchen, the bathroom etc.). We made use of the inherent elements and properties of the HE for the classification of these three dimensions. The HE comprises a checklist of 161 environmental barrier items [10]. Each barrier item has a descriptive label attached, designating barrier characteristics, such as “Paths narrower than 1.5 m”, “High thresholds at entrance more than 15 mm” etc. Further, for each barrier item there is a 14-position scoring pattern, where the scores denote severity of accessibility problems related to 14 functional limitations of the individual. Table 1 shows the 14 functional limitations of the HE that the scoring patterns are related to.

**Table 1:** Functional limitations/use of mobility devices included in the 14 position scoring pattern of the HE^a^

Functional limitation	Position of scoring pattern
Difficulty in interpreting information	1
Severe loss of sight	2
Complete loss of sight	3
Severe loss of hearing	4
Prevalence of poor balance	5
Incoordination	6
Limitations of stamina	7
Difficulties in moving head	8
Difficulty in reaching with arms	9
Difficulty in handling and fingering	10
Loss of upper extremity skills	11
Difficulty in bending, kneeling, etc.	12
Reliance on walking aids	13
Wheelchair user	14

^a^The Housing Enabler instrument [10]

**Fig. 1:**
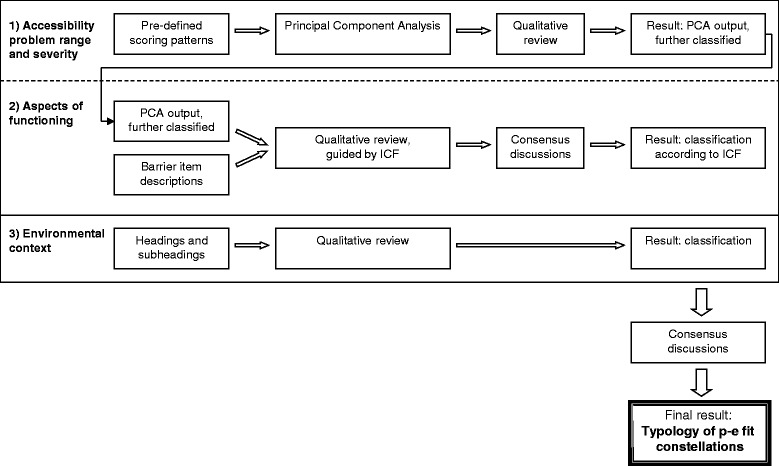
Flowchart showing the classification procedures used in order to construct the typology of person-environment fit constellations. For more details of the generation of the pre-defined scoring patterns, see [9], and for an overview of the continuous validation process, see [13]

The scoring positions of the patterns are graded from 0 to 4 (0 = no problem, 1 = potential problem, 2 = problem, 3 = severe problem, 4 = impossibility). When constructing the typology the scoring patterns and the pool of the 161 environmental barrier specifications constituted the data to be classified. That is, we did not use any empirical data but only the content and scoring system of the HE instrument. For an overview of how we proceeded to construct the typology, see Fig. 1.

### 1/ Classification of the accessibility problem range and severity

To classify the accessibility problem range (i.e., based on the functional limitations involved) and severity (i.e., as indicated by the scoring patterns), we first applied a statistical approach using Principal Component Analysis (PCA) (see e.g. [18, 19]) to the pre-defined severity scores of the HE instrument. We thus ran a PCA on the scoring patterns of the 161 environmental barrier items, that is, the bivariate between-barrier correlations of the barriers’ 14 severity scores. In simple terms we thereby aimed to identify groups of similar scoring patterns inherent in the HE. PCA is well suited for this task, as it basically adopts a purely data descriptive algorithm, which decomposes the overall covariation of the scoring patterns comprised in the correlation matrix into unique orthogonal components and does not imply distributional assumptions. Notably, PCA is based on a kind of grouping of the items analyzed according to their similarity in terms of correlation. A high correlation of the scores assigned to each of a given pair of environmental barriers means that they have a similar pattern of range and severity in generating accessibility problems across the 14 functional limitations. That is, both environmental barriers would provide relatively high or low risks of accessibility problems across the same functional limitations. Note however, that this does not necessarily mean that they generate problems of equal severity, as we did not intend to analyze the environmental barriers due to the absolute equality of the pre-defined severity scores. Hence, the loadings obtained from the Varimax rotated factor solution (for computational details, see [18]) was used to group barriers with similar patterns of their scores across the 14 functional limitations. Each group of similar scoring patterns thus constitutes a class of this dimension of the classification system.

Though this statistical approach provided an initial classification, it was considered to need an additional qualitative review, as the component output, by the inherent rationale of the procedure, would not be distinct enough to to be valid for our particular purpose. That is, in statistical terms the similarities in a typology should demonstrate minimum within-group variance and maximum between-group variance [20], which is not entirely the case regarding the PCA output. Therefore we proceeded in a second, complementary analytic step. Principal components that included scoring patterns which we considered as too varied were further subdivided by means of qualitative review. The classification was finalized during consensus discussions.

Further, in order to achieve the unified scoring patterns needed for generalizability, we made a final balancing of the original scoring patterns included. For example, if there were five scoring patterns included in a class and three of them had a severity grade 2 for a certain position, while the others had a severity grade 3, we needed to choose between these. This was done by calculating the mean scores (rounded to integer values) for each position of the scoring patterns. Mean scores were chosen because they designate minimum deviance (i.e., variance) of the scoring patterns subsumed.

### 2/ Classification of the aspects of functioning

Since components of functioning and disability such as “Body Function and Structures”, “Activities and Participation”, “Environmental Factors” and “Personal Factors” are relevant in this context, we decided to use the International Classification of Functioning, Disability and Health (ICF) [21] as a conceptual framework. The underlying idea of problematic person-environment fit constellations is the assumption that environmental barriers as such do not generate accessibility problems, but may do so when connected to certain aspects of physical functioning [22]. For instance, high kerbs may constrain walking and too high/low seats may constrain sitting for people with difficulties bending and kneeling, and hence generate accessibility problems. In other words, the accessibility problems are specific to an individual; what hinders one person might not be an issue for someone else.

The classification of aspects of functioning was carried out in an iterative process, by means of qualitative review and consensus discussions. Four researchers with documented practical and scientific experience of accessibility assessments and methodological development were engaged (see e.g. [13, 23–25]). In the first step of the process one of the researchers reviewed a list where the 161 barrier items were sorted according to the PCA output (as described above). Doing so aided the classification of aspects of functioning, as similarities in scoring patterns often, though not always, imply similar functioning. The guiding classification principle was to find the ICF classification that most closely corresponded to the aspect of functioning implied by the barrier specification, together with the functional limitations involved, as indicated by the scoring patterns. If more than one aspect of functioning was involved, the predominant aspect―in terms of severity―was noted first in order. Following this step, the other researchers reviewed the proposed classification, and suggested alterations and adjustments. After repeated consensus discussions, the classification was finalized when all classifications were agreed upon.

### 3/ Classification of the environmental contexts

Classification by environmental contexts was done in parallell and independently of the other two dimensions. Although the ICF also considers environmental factors as something external that may influence the individual’s capacity [21], the categories specified therein are not related to detailed features of the built environment. That is why the ICF was not used for this dimension. Instead, we basically used the headings and subheadings from the HE instrument manual to classify the environmental barriers according to environmental context. For reasons of parsimony some headings were merged (such as entrance doors and ramps) when forming the classes. The HE checklist of barriers in the housing environment is to some extent general with respect to the environmental context in which the barriers occur. Barriers such as the width of door openings, insufficient maneuvering space, controls placed too high or too low, etc., contain features that evidently occur also in other environmental arenas [16].

### The typology of person-environment fit constellations

Finally, the typology of person-environment fit constellations was constructed by combining the classifications of all three dimensions. The groups with common characteristics thus generated are the types of the typology [26]. In this study the types are the particular combinations of aspects of functioning as related to the environmental contexts that generate accessibility problems of a certain range and severity. The types were sorted according to the ICF framework, and labelled by the order in which they appear in the typology, beginning with T1.

## Results

By use of the PCA we first arrived at 13 principal components (as each barrier got scores for 14 functional limitations), that accounted for the total variation of the scores across the functional limitations. After further subdivision and final balancing of the scoring patterns included in these 13 initially identified principal components, the classification of accessibility problem range and severity (as quantified by the scoring patterns) ended up with 48 different classes, labeled T1 to T48. Five of these classes of scoring patterns covered ten or more environmental barriers, while fifteen covered just one.

As a result of the classification according to the ICF, five different blocks of functioning―all under the “Activity and Participation” component of the ICF―were recognized as predominant. They represented “Learning and Applying Knowledge”, “Communication” and “Mobility”, either alone or in combination. “Purposeful sensory experiences” was predominant for types T1-T4, “Applying knowledge” for T5-T6, “Changing and maintaining body position” for T7-T17, “Carrying and handling objects” for T18-T33 and “Walking and moving” for T34-T48. Three additional ICF blocks of functioning occured as subordinated. In total the functioning blocks covered 28 different functioning categories. The classification of the environmental context dimension resulted in nine different classes. The contexts with the highest frequencies of environmental barrier items were “Hygiene area” (28 items), “Kitchen/Laundry/Utility kitchen” (24 items) and “Stairs” (22 items). Table 2 gives an overview of the 48 types with regard to the two dimensions aspects of functioning and environmental contexts.

**Table 2:** Overview of the 48 types with regard to aspects of functioning and environmental context, as they are represented in the HE checklist for the housing environment. The types are listed by their predominant aspect of functioning

		Environmental context^a^								
Type	Aspect of functioning, ICF block^b^	Paths & roadway/seating places	Parking	Entrance door/ramps	Stairs	Lifts	Hygiene area	Kitchen/laundry/utility kitchen	Indoor, except hygiene/kitchen	Sitting out place/balcony/suppl housing facilities
T1 - T4	Purposeful sensory experiences	X	(X)	X	X	X	(X)	X	X	(X)
T5 - T6	Applying knowledge	(X)	(X)	X	(X)	X	X	X	X	
Subordinated	Communicating, receiving					(X)	(X)	(X)	(X)	
Subordinated	Conversation and use of communication devices			(X)						
T7 - T17	Changing and maintaining body position	X		(X)	X	X	X	X	X	X
T18 - T33	Carrying, moving and handling objects			X		X	X	X	X	X
T34 - T48	Walking and moving	X	X	X	X	X	X	X	X	X
Subordinated	Moving around using transportation		(X)							
	Total number of occurrences in the HE^c^	19	5	15	22	16	28	24	20	12

Note: crossmark in parenthesis indicates occurrence only where aspect of functioning is subordinated

^a^Basically headings/subheadings from the Housing Enabler instrument [10]

^b^International Classification of Functioning, Disability and Health [21]

^c^ *N* = 161 items

The functioning blocks “Walking and moving” and “Purposeful sensory experiences” were represented in all environmental context classes. “Applying knowledge” and “Changing and maintaining body position” were represented in all classes except “Sitting out place/balcony/supplementary housing facility” and “Parking”, respectively.

Combining all three dimensions into a typology highlights their complex internal relations. In Table 3 the typology of the 48 typical person-environment fit constellations is provided, and each type is exemplified with an environmental barrier of the HE.

**Table 3:** Typology of 48 typical person-environment fit constellations, with the ICF as conceptual framework and scoring patterns that quantify range (i.e. functional limitations/use of mobility devices involved) and severity of accessibility problems

Type	Accessibility problem	Aspect of functioning^b^		Environmental context	Environmental barrier example^c^	No of items
	Scoring pattern^a^	ICF block	ICF category			
T1	04000000000000	Purposeful sensory experiences	Watching	Stairs Kitchen/laundry room/utility kitchen	Poor illumination of walking area and/or handrails.	3
T2	13000000000000	Purposeful sensory experiences/Applying knowledge	Watching/Focusing attention	Paths and roadways/Seating places Stairs	Visual pattern on the surface of stair treads camouflages edges of treads.	5
T3	13300010000011	Purposeful sensory experiences/Applying knowledge/Walking and moving	Watching/Focusing attention/Walking short distance	Entrance door/ramps Lifts Kitchen/laundry room/utility kitchen Paths and roadways/Seating places Indoor, except hygiene/kitchen Entrance door/ramps Stairs	Complicated/illogical routes to/from entrance.	9
T4	00030030000011	Purposeful sensory experiences/Changing and maintaining body position	Listening/Maintaining a standing position	Lifts	No visual signal when the lift arrives.	1
T5	42300000000000	Applying knowledge/Purposeful sensory experiences/Communicating, receiving/	Applying knowledge, other specified/Watching/Non-verbal messages	Lifts Kitchen/laundry room/utility kitchen Hygiene area Indoor, except hygiene/kitchen	Illogical design of controls.	4
T6	33300300011000	Applying knowledge/Purposeful sensory experiences/Use of communication devices/Carrying, moving and handling objects	Applying knowledge/Watching/Using communication device Manipulating	Entrance door/ramps Kitchen/laundry room/utility kitchen Hygiene area Indoor, except hygiene/kitchen	Complicated/illogical opening procedure. Also includes entry phone.	4
T7	00000003000300	Changing and maintaining body position	Standing	Kitchen/laundry room/utility kitchen	Low working surfaces	1
T8	00000030000003	Changing and maintaining body position	Sitting/Standing	Hygiene area	Wash-basin placed at a height for use only when standing	2
T9	00000000000010	Changing and maintaining body position	Maintaining a standing position	Hygiene area	Grab bars in low position.	1
T10	00000000000110	Changing and maintaining body position	Maintaining a standing position	Kitchen/laundry room/utility kitchen Hygiene area Indoor, except hygiene/kitchen	Controls in low position	3
T11	00002010000023	Changing and maintaining body position	Sitting/Standing/Maintaining a sitting/standing position/	Kitchen/laundry room/utility kitchen Hygiene area Sitting out place/balcony/suppl housing	No surface at a height suitable for sitting while working.	5
T12	00001000000332	Changing and maintaining body position	Sitting/Standing/Maintaining a sitting/standing position	Hygiene area	Insufficient space for stool, bath board, or equivalent, or other problem in shower/bath.	3
T13	00000000000001	Changing and maintaining body position	Transferring oneself while sitting	Hygiene area	Toilet 48 cm or higher. Including seat.	2
T14	00100101210000	Changing and maintaining body position/Purposeful sensory experience	Maintaining a sitting position/Watching	Hygiene area	Toilet roll holder in inaccessible position	1
T15	11103330000141	Changing and maintaining body position/Purposeful sensory experiences/Applying knowledge	Maintaining a standing position/Watching/Focusing attention	Hygiene area Stairs	No grab bar at shower/bath and/or toilet.	3
T16	00003230000030	Changing and maintaining body position/Walking and moving	Maintaining a standing position Walking	Lifts Paths and roadways/Seating places	No seat in lift.	2
T17	00001110000010	Changing and maintaining body position/Walking and moving	Maintaining a sitting/standing position/Climbing stairs	Stairs Lifts Paths and roadways/Seating places	Handrails placed too high/low	8
T18	00000000320000	Carrying, moving and handling objects	Carrying in the hands	Kitchen/laundry room/utility kitchen	Hobs with ordinary rings. Also includes gas stoves, coil stoves, etc.	1
T19	00000000024000	Carrying, moving and handling objects	Grasping/Manipulating/Releasing Turning or twisting	Kitchen/laundry room/utility kitchen Hygiene area Indoor, except hygiene/kitchen Lifts	Use requires fingers (i.e. isolated grip, e.g. pinch and lateral grip).	10
T20	00000000034010	Carrying, moving and handling objects	Hand and arm use, other specified	Kitchen/laundry room/utility kitchen Hygiene area Indoor, except hygiene/kitchen	Use requires hands.	3
T21	02200000032000	Carrying, moving and handling objects/Purposeful sensory experiences	Manipulating/Turning or twisting/Watching	Kitchen/laundry room/utility kitchen Hygiene area Indoor, except hygiene/kitchen	Very small controls.	3
T22	01101101300013	Carrying, moving and handling objects/Purposeful sensory experiences/Changing and maintaining body position	Reaching/Watching/Maintaining a sitting/standing position	Kitchen/laundry room/utility kitchen Hygiene area Indoor, except hygiene/kitchen	Inappropriate design of wardrobes/clothes cupboards.	2
T23	03304332434334	Carrying, moving and handling objects/Purposeful sensory experiences/Changing and maintaining body position	Reaching/Watching/Maintaining a sitting/standing position	Kitchen/laundry room/utility kitchen	Wall-mounted cupboards and shelves placed high	1
T24	10000300010010	Carrying, moving and handling objects/Applying knowledge	Grasping/Manipulating/Releasing/Focusing attention	Kitchen/laundry room/utility kitchen Hygiene area Indoor, except hygiene/kitchen	Use requires intact fine motor control.	3
T25	20100200011010	Carrying, moving and handling objects/Applying knowledge	Grasping/Manipulating/Releasing/Applying knowledge, other specified	Kitchen/laundry room/utility kitchen Hygiene area Indoor, except hygiene/kitchen	Complex manoeuvres (more than one operation/movement) and good precision required.	3
T26	10000200014030	Carrying, moving and handling objects/Applying knowledge	Hand and arm use, unspecified/Focusing attention	Kitchen/laundry room/utility kitchen Hygiene area Indoor, except hygiene/kitchen	Use requires two hands.	3
T27	00000002311024	Carrying, moving and handling objects/Changing and maintaining body position	Reaching/Maintaining a sitting/standing position	Lifts Kitchen/laundry room/utility kitchen Hygiene area Indoor, except hygiene/kitchen	Controls placed too high/low. Refers to both outside and inside the lift	5
T28	00000030010031	Carrying, moving and handling objects/Changing and maintaining body position	Grasping/Manipulating/Maintaining a body position, other specified	Kitchen/laundry room/utility kitchen Hygiene area Indoor, except hygiene/kitchen	High force required to activate controls.	3
T29	00003330304033	Carrying, moving and handling objects/Changing and maintaining body position	Pushing/Pulling/Maintaining a sitting/standing position	Entrance door/ramps Lifts	Heavy doors without automatic opening.	2
T30	00000001004414	Carrying, moving and handling objects/Changing and maintaining body position	Pushing/Pulling/Maintaining a sitting/standing position	Kitchen/laundry room/utility kitchen	Door swings (inner doors) which impede accessibility to storage units.	1
T31	00003210334034	Carrying, moving and handling objects/Changing and maintaining body position	Reaching/Maintaining a sitting/standing position	Sitting out place/balcony/suppl housing	Refuse bin difficult to reach	2
T32	00000000404333	Carrying, moving and handling objects/Changing and maintaining body position	Reaching/Maintaining a sitting/standing position	Kitchen/laundry room/utility kitchen	Shelves too deep. Deeper shelves require pullout shelves/turntable units.	1
T33	00001120300211	Carrying, moving and handling objects/Changing and maintaining body position	Reaching/Maintaining a sitting/standing position	Hygiene area	Grab bar difficult to reach/inappropriately positioned (NOT as regards height).	1
T34	00000000000034	Walking and moving	Moving around using equipment	Paths and roadways/Seating places Entrance door/ramps Lift Sitting out place/balcony/suppl hous Indoor, except hygiene/kitchen	Insufficient manoeuvring space at seating places.	10
T35	03303301000034	Walking and moving/Purposeful sensory experiences	Walking/Moving around using equipment/Watching	Paths and roadways/Seating places Sitting out place/balcony/suppl housing Entrance door/ramps Lifts Indoor, except hygiene/kitchen Kitchen/laundry room/utility kitchen	Irregular/uneven surface (irregular surfacing, joins, sloping sections cracks, holes).	12
T36	03300040000012	Walking and moving/Purposeful sensory experiences	Walking/Moving around using equipment/Watching	Parking	Passenger loading zones far from entrance.	1
T37	01103333000034	Walking and moving/Purposeful sensory experiences	Walking/Moving around using equipment/Watching	Parking Paths and roadways/Seating places	No stable, even, non-slip surface in car park (loose gravel, sand, clay, etc.).	2
T38	01100000000030	Walking and moving/Purposeful sensory experiences	Climbing stairs/Moving around using equipment/Watching	Stairs	Projecting nosing/open-riser stairs.	1
T39	03300000000033	Walking and moving/Purposeful sensory experiences	Moving around using equipment/Watching	Paths and roadways/Seating places Entrance door/ramps	Furniture placed in the path of travel.	2
T40	01100000000014	Walking and moving/Purposeful sensory experiences	Moving around using equipment/Watching	Entrance door/ramps	Door swing that obstructs use. Refers to door leaves that obtrude when opening and/or closing.	1
T41	23403330000033	Walking and moving/Purposeful sensory experiences/Applying knowledge	Walking/Moving around using equipment/Watching/Applying knowledge, other specified	Entrance door/ramps Lift	Doors that do not stay in open position/close quickly.	2
T42	13303330000032	Walking and moving/Purposeful sensory experiences/Applying knowledge	Walking/Climbing stairs/Moving around using equipment/Watching/Applying knowledge, other specified	Paths and roadways/Seating places Stairs	Routes with steps.	11
T43	12012001000033	Walking and moving/Purposeful sensory experiences/Applying knowledge	Walking/Moving around using equipment/Watching/Focusing attention	Paths and roadways/Seating places	Poor/uneven/dazzling lighting along circulation paths.	1
T44	13302303000033	Walking and moving/Purposeful sensory experiences/Applying knowledge	Walking/Moving around using equipment/Watching/Focusing attention	Lifts	Wide gap between the lift and the building floor.	1
T45	30000003000033	Walking and moving/Applying knowledge	Moving around using equipment/Applying knowledge, other specified	Parking	No marked parking for people with functional limitations within 10 m of the entrance.	1
T46	40001140000012	Walking and moving/Applying knowledge	Walking/Applying knowledge, other specified	Parking	Parking place far from entrance.	1
T47	00000200000034	Walking and moving/Changing and maintaining body position	Moving around using equipment/Maintaining a body position, other specified	Indoor, except hygiene/kitchen Hygiene area	Insufficient manoeuvring spaces where turning is necessary.	2
T48	00003330000033	Walking and moving/Moving around using transportation/Changing and maintaining body position/Carrying, moving and handling objects	Walking/Using transport/Moving around using equipment Maintaining a standing position/Climbing stairs/Pushing/Pulling	Paths and roadways/Seating places Entrance door/ramps Sitting out place/balcony/suppl housing Lifts Parking	Rough/unstable ground at seating places	11

Note: a type is defined by the combination of characteristics of a particular person-environment fit constellation

^a^For functional limitations related to each position of the scoring pattern, see Table 1. Severity grades from 0 (=no problem) to 4 (=impossibility)

^b^International classification of functioning, disability and health [21]

^c^Environmental barrier items from the Housing Enabler checklist for the housing environment [10]

**Fig. 2:**
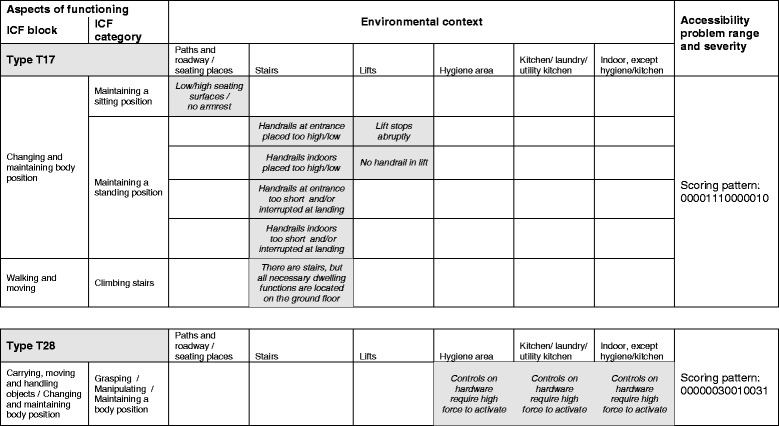
Examples from the typology of individual manifestations of person-environment fit constellation in the housing environment, types T17 and T28. For functional limitations related to each position of the scoring pattern, see Table 1. Severity grades from 0 (=no problem) to 4 (=impossibility). Note: a type is defined by the combination of characteristics of a particular person-environment fit constellation

Most of the types, even those that cover ten or more environmental barrier items, showed a high degree of homogeneity in the aspects of functioning implied. However, many types were represented in several environmental contexts, as exemplified in Fig. 2. Eight environmental barrier items were covered by T17, occuring in three different environmental contexts. Accessibility problems generated by T17 all have the severity grade scored 1, related to “Poor balance”, “Incoordination”, “Limitations of stamina” and “Reliance on walking aids” (positions 5, 6, 7 and 13 of the scoring pattern). That is, several functional limitations are involved, but the severity grade is low. For T28 three items were covered, all identical in the aspects of functioning implied, but each recognized in a different environmental context. Accessibility problems scored 3 in T28 were related to “Limitations of stamina” and “Reliance on walking aids” (positions 7 and 13 of the scoring pattern), while accessibility problems scored 1 were related to “Difficulty in handling and fingering” and “Wheelchair use “(positions 10 and 14 of the scoring pattern).

## Discussion

By the construction of a typology of person-environment fit constellations we have provided a novel platform for further studies and instrument development within the field of accessibility research. This was achieved by classifying the inherent elements and properties of an existing instrument targeting housing accessibility. Similarities as well as differences in how typical accessibility problems are manifested as physical barriers in the environment are made more easily recognized.

As evidenced by Table 3, the typology of person-environment fit constellations covers accessibility problems of a varied range and severity, within the ICF framework stretching from “Purposeful sensory experiences” to “Moving around using equipment. Even though the housing environment―constituting only one out of several different environmental arenas where people live their lives―can not be presumed to cover all possible person-environment fit constellations that creates accessibility problems, the typology provides a basis for the extension of the EM to other environmental arenas. The scoring of accessibility problems, generated by juxtaposing each environmental barrier with each functional limitation, is thereby connected to general person-environment fit constellations. Hence, when creating new checklists for other environmental arenas, providing the severity scores is ideally only a matter of finding the proper classification. By tracing which aspects of functioning that can be related to the new barriers, it should be possible to recognize similarities and find existing scoring patterns that are already present in the typology.

A practical example of the relevance and usability of the typology might be that an instrument applicable for assessing the accessibility of entrances at public facilities is sought for. First environmental barriers in that context should be determined, for instance “Visual information signs that are difficult to read at an appropriate distance”. Next, the implied aspects of functioning in terms of the ICF classification should be identified, in this case “Purposeful sensory experiences” and “Applying knowledge”. Finally, by scrutinizing aspects of functioning, range and severity of the accessibility problem and with the help of existing examples of environmental barriers given in the typology, an appropriate scoring pattern can ideally be established. In this example, “Purposeful sensory experiences” and “Applying knowledge” are predominant aspects in types T2-T3 and T5-T6. The barrier in question does not relate to “Walking and moving”, which is included in T3; it might relate to “Communicating, receiving” but is not related to “Blindness” which is included in the scoring pattern of T5. The barrier is neither related to “Use of communication devices”, nor to “Carrying, moving and handling objects” as included in T6, which means that the most appropriate of the existing scoring patterns seems to be that of T2. However, the reliability of this procedure as well as the validity of the scoring patterns identified for new environmental barrier items remain to be tested.

Likewise, the typology allows for scanning of contexts to which environmental barriers of a particular type are concentrated. This is exemplified in Fig. 2, where five out of eight T17 barriers are concentrated to stairs. The same kind of illustrations as exemplified in Fig. 2 can be provided for all 48 types, thus providing a full overview. Accordingly, the typology may serve as an inventory tool of problematic person-environment fit constellations with the potential to aid future instrument development useful for accessibility research.

### Study limitations

The way we used the ICF calls for a comment. Environmental factors according to the ICF embrace a broad range of aspects that make up the physical, social and attitudinal environment in which people live their lives, from the immediate environment to overarching systems in the society, such as infrastructure and policies [21]. That is, the physical environment in terms of the natural and built environment is not described in sufficient detail. For a comprehensive understanding of how disability is generated by the interaction of environmental factors and the individual however, more detailed knowledge of the environment is needed [27]. By considering the environmental barrier specifications of the HE together with the range and severity of accessibility problems, information on the interaction of the environment and the individual was gained and made the classification process feasible. Even though our approach is not consistent with the procedure for linking items to the ICF as described by others (see e.g. [28]), using the ICF as a guiding conceptual framework contributed to the development of further knowledge on how health-related domains are related to environmental barriers and the subsequent generation of accessibility problems.

The choice of PCA as the statistical tool for the classification of accessibility problem range and severity (quantified as scoring patterns), also needs to be discussed. As an alternative analytic approach we considered Cluster Analysis (CA) [18], which is traditionally recommended for classification purposes (see e.g. [17]). In contrast to PCA, CA implies a definition of similarity in terms of the absolute values of the scores, meaning that two environmental barrier items are similar to the degree of equality of their scores. However, this implicit concept of similarity may be too restrictive with respect to the overarching analytical aim of the present study. This expectation was confirmed when we initially run CA to test its feasibility for our purpose, trying out several hierarchical clustering algorithms which altogether did not reveal any clear-cut cluster solution. Thus, it seems that the environmental barriers of the HE are not strictly clustered into few groups according to their scores across the 14 functional limitations. In addition, it should also be considered that the limited score range (0 to 4) means that if the scoring patterns of two environmental barriers are highly correlated, the pair-wise differences between the scores cannot be too large, implying also some degree of similarity in terms of absolute values of the scores. If so, CA would not provide at all much additional benefit compared with PCA.

The proposed typology may appear to be too specific as many of the presumed typical constellations only cover one environmental barrier item. However, any typology is bound to be arbitrary to some extent, as there is a need to select a limited number of attributes from a universe of possible choices [20, 29]. The attributes chosen must strike a balance between being too few and therefore too general, versus being too many and thereby too specific. Moreover, it has to be kept in mind that the typology so far is based on an instrument specifically targeting the housing environment. For example, the constellation that in this typology is only manifested by the absence of visual signal when the lift arrives concerns an environmental barrier that obstructs access in relation to loss of hearing. In public environmental arenas, there are probably many more manifestations of environmental barriers obstructing access in relation to loss of hearing that would be covered by this constellation [30]. Thus, this is an example that demonstrates the potential of the typology to be useful for efforts that aim to extend the EM to other environmental arenas.

Yet, the typology proposed represents a first exploratory step and further methodological research is needed before it can be validly used in various environmental arenas. There are also considerable opportunities for improvement of visual guidelines and display of the typology, including to give the types more meaningful labels in order to facilitate its use as inventory of person-environment fit constellations.

## Conclusions

The main contribution of this study is the proposed typology of person-environment fit constellations, based on an existing, internationally acknowledged instrument for assessment and analyses of housing accessibility. Besides being an elegant solution for the extension of the EM to other environmental arenas, it provides a novel methodological platform for the identification and quantification of problematic person-environment fit constellations. Its link to the globally accepted ICF classification system is an advantage, as it facilitates communication within the scientific and health care practice communities. The typology has the advantage of reducing the complexity of reality into a simplified and structured scheme, thus rendering similarities as well as differences more easily detectable. The elucidation of the relations between the dimensions of physical functioning, environmental contexts and scoring patterns contributes to the knowledge base necessary to advance in the field of accessibility research. Since it nurtures reflections on the interaction of functional limitations and environmental barriers in different contexts, the typology furnishes a reference point for further research, ultimately aiming for an environment supportive of activity, participation and health for all.

## Abbreviations

CA: Cluster analysis
EM: Enabler methodology
ETA: Ecological theory of ageing
HE: Housing enabler
ICF: The International classification of functioning, disability and health
PCA: Principal component analysis
WHO: World Health Organisation
